# Radiation-Related Fear, Risk Perception, and Occupational Responses Among Healthcare Workers and Health Professions Students: A Scoping Review

**DOI:** 10.3390/healthcare14193353

**Published:** 2026-10-08

**Authors:** Venera M. Almatova, Zhanar Kozhakhmetova, Indira M. Bozheyeva, Gaukhar L. Medetova, Karlygash S. Absattarova, Karlygash K. Toguzbaeva

**Affiliations:** 1 Scientific Research Institute of Radiology Named After Academician Zh. H. Khamzabayev, Astana Medical University, Astana 010000, Kazakhstan; almatova.v@amu.kz (V.M.A.); zhanar5@mail.ru (Z.K.); bozheeva.i@amu.kz (I.M.B.); medetova.g@amu.kz (G.L.M.); 2 Department of Public Health, S. D. Asfendiyarov Kazakh National Medical University, Almaty 050012, Kazakhstan; toguzbaeva@gmail.com

**Keywords:** radiophobia, ionising radiation, healthcare workers, health professions students, risk perception, occupational anxiety, radiation protection, scoping review

## Abstract

**Highlights:**

**What are the main findings?**

- Professional radiophobia is operationalised inconsistently; the evidence does not support a single pooled prevalence estimate.
- Knowledge, experience, perceived exposure, dosimetry, equipment usability, and safety climate were reported alongside variation in concern and protective behaviour.

**What are the implications of the main findings?**

- Practice-oriented radiation-safety strategies may need to address dose literacy together with practical training, personal monitoring, usable protective equipment, and transparent risk communication.
- Future studies require validated multidimensional instruments separating knowledge, risk appraisal, affective anxiety, trust, protection, and avoidance.

**Abstract:**

**Background/Objectives:** Radiation-related fear, anxiety, risk perception, reproductive concern, protective behaviour, and avoidance are conceptually related but not interchangeable. This scoping review mapped how these constructs have been operationalised among healthcare workers and health professions students. Methods: Following JBI guidance and PRISMA-ScR, PubMed, Scopus, Web of Science Core Collection, and the Cochrane Library were searched for English-language records published from 1 January 2000 to 4 September 2026. Of 845 records, 388 duplicates were removed and 457 unique records were screened. **Results:** Twenty-seven reports underwent full-text assessment; one was excluded because its mixed population and radiation context did not permit a healthcare-specific occupational interpretation, leaving 26 studies. The evidence was dominated by cross-sectional studies and heterogeneous measurement instruments; therefore, study-level percentages were not treated as directly comparable prevalence estimates. Reported outcomes ranged from explicit radiophobia and affective anxiety to cognitive risk appraisal, reproductive concern, protective behaviour, and avoidance. Education was associated with differing changes across settings, while dosimetry, protective equipment, workflow, and safety climate were reported alongside occupational responses. **Conclusions:** The literature does not support a standardised operational definition or common measurement framework for professional radiophobia. Future research should distinguish affective fear or anxiety from contextually appropriate occupational risk appraisal and protective behaviour, and should evaluate organisational conditions alongside education.

## 1. Introduction

Medical ionising radiation is indispensable for diagnosis and treatment, while occupational protection is governed by justification, optimisation, and dose limitation [1,2]. These principles address exposure control, but they do not by themselves explain how workers interpret uncertain or low-dose risks. Radiation is invisible, possible effects may be delayed, and quantities such as effective dose are not intuitively understood. Consequently, objectively controlled exposure can coexist with substantial concern.

Professional radiophobia has no accepted diagnostic or operational definition. Across the literature, the term has been used alongside explicit fear, anxiety, heightened perceived risk, concern about cancer or reproductive effects, and avoidance of radiation-related work. These constructs are related but not interchangeable: affective fear or anxiety concerns emotional response; perceived radiation risk concerns cognitive appraisal of probability or severity; reproductive concern concerns fertility, pregnancy, foetal, or future-child effects; protective behaviour concerns dose-reduction actions; and avoidance concerns reduction, withdrawal, or refusal of radiation-related activity because of concern. A low knowledge score is not evidence of phobia, and use of a dosimeter or protective equipment can represent appropriate safety behaviour rather than fear. For this review, these constructs were treated as analytically distinct rather than as severity levels on a single continuum, and the term radiophobia was retained only where the source itself used it or directly operationalised fear in those terms.

The psychometric paradigm identifies unfamiliarity, involuntariness, delayed consequences, and limited perceived control as characteristics that intensify risk perception [3]. Ethical radiological protection also requires transparency, accountability, dignity, and stakeholder involvement [4]. In healthcare settings, these considerations extend beyond individual emotion because concern may influence participation in procedures, career decisions, communication with patients, and adherence to protection practices.

Previous systematic reviews addressed related but narrower questions. Behzadmehr et al. [5] synthesised knowledge, attitudes, and practices regarding radiation protection among healthcare workers, whereas Rodrigues et al. [6] focused on radiation-protection literacy and knowledge among exposed healthcare professionals. Neither review was designed primarily to map affective fear or anxiety, reproductive concern, risk appraisal, avoidance, and organisational responses as distinct constructs. The present scoping review therefore aimed to (1) map definitions and measures of radiophobia and related radiation concern; (2) distinguish affective, cognitive, reproductive, behavioural, and organisational domains; (3) describe reported associations with knowledge, experience, demographic factors, protective behaviour, and organisational conditions; and (4) identify implications for measurement, education, and occupational-radiation protection. The operational definitions used to organise the evidence map are summarised in Table 1.

## 2. Materials and Methods

### 2.1. Design and Reporting

The review followed JBI methodological guidance for scoping reviews [7] and was reported with reference to PRISMA-ScR [8] and the PRISMA 2020 flow structure [9]. A scoping design was chosen because the literature used heterogeneous populations, concepts, instruments, and study designs. The protocol was not prospectively registered because public registration was not considered mandatory for a scoping review when the study was initiated. The exact date on which the review methods were finalised was not prospectively recorded, and no publicly accessible archived protocol or review plan is available. The methods described below reflect the predefined approach used for the review.

### 2.2. Review Question and PCC Framework

The review question was: How have radiophobia, radiation-related fear, anxiety, concern, and risk perception been measured among healthcare workers and health professions students in occupational or clinical-training contexts, and what factors and behaviours have been reported in association with these outcomes? Population comprised healthcare workers and students preparing for healthcare roles. The concept comprised explicit radiophobia or radiation-related fear, anxiety, concern, risk perception, or fear-linked avoidance. The context comprised occupational healthcare practice or clinical training involving ionising radiation.

### 2.3. Information Sources and Search

PubMed was searched on 3 September 2026; Scopus, Web of Science Core Collection, and the Cochrane Library were searched on 4 September 2026. Searches covered English-language records published from 1 January 2000 to the respective search date. The strategy combined terms for radiophobia, fear, anxiety, concern, and radiation-risk perception with terms for healthcare personnel and health professions students. Complete database-specific strategies, limits, dates, and database-reported yields are presented in Supplementary Table S1. These databases were selected to cover biomedical, multidisciplinary, and evidence-synthesis literature. Resource constraints precluded Embase, PsycINFO, CINAHL, and a structured grey-literature search; consequently, studies indexed exclusively in dedicated nursing, psychological, or other subject-specific databases may have been missed. Formal backward reference-list screening, forward citation tracking, and independent peer review of the search strategy were not documented and were therefore not claimed. The final Scopus RIS and CSV exports each contained 359 records; an earlier partial 90-record Scopus export was an intermediate subset and was not counted separately. For studies published in 2026, publication year was assigned according to the bibliographic publication record, including online-first publication where applicable. The included 2026 reports were published online before the final search dates; however, exact database-indexing dates were not retained and cannot be reconstructed from the available search records.

### 2.4. Eligibility Criteria

Eligible reports were original quantitative, qualitative, or mixed-methods studies involving healthcare workers or health professions students that operationalised at least one substantive ionising-radiation outcome within an occupational or clinical-training context: radiophobia; radiation-related fear; anxiety or worry; occupational or reproductive concern; perceived radiation risk; protective behaviour linked to concern; or avoidance attributable to concern. Risk perception alone was sufficient for eligibility when it was a substantive ionising-radiation outcome in the relevant occupational or training context; it was not assumed to be equivalent to fear or anxiety. Studies measuring knowledge, awareness, or protection compliance alone were excluded unless these were analysed alongside an eligible affective, cognitive risk-perception, reproductive, or concern-linked behavioural outcome. Studies restricted to patients, the general public, industrial workers, non-ionising radiation, or non-clinical nuclear-accident response were excluded, as were reviews, editorials, conference abstracts without sufficient data, and unverifiable reports.

### 2.5. Selection and Data Charting

The four database result sets were combined and deduplicated using exact DOI and normalised-title matching, with manual checks for ambiguous matches. The recorded retrieval comprised 845 records: PubMed 235, Scopus 359, Web of Science Core Collection 243, and Cochrane Library 8. Deduplication removed 388 records (355 exact DOI matches and 33 exact normalised-title matches), leaving 457 unique records. One reviewer screened all titles and abstracts and assessed potentially eligible full texts; a second reviewer checked potentially eligible records and full-text eligibility decisions. Disagreements were resolved through discussion and consensus. The exact proportion of records independently checked and the number of disagreements were not prospectively recorded.

One primary reason was recorded for each full-text exclusion. Data were charted by country, population, design, sample, instrument or construct, main findings, and limitations affecting interpretation. One reviewer initially charted the data using a structured form. No formal pilot-testing or calibration phase of the charting form was documented, and study investigators were not contacted; extraction relied on the published reports and accessible supplementary materials. Following peer review, a second author independently rechecked the key numerical and descriptive extractions for all 26 included studies against the original reports, and source-level inconsistencies were retained rather than silently recalculated. The resulting verification dataset is provided in Supplementary Table S4. Funding sources of the included studies were not systematically charted.

### 2.6. Synthesis and Critical Interpretation

Because outcome definitions and measurement scales were not commensurable, no meta-analysis or pooled prevalence estimate was undertaken. For narrative and framework synthesis, the charted findings were organised into analytically distinct domains: direct fear or source-labelled radiophobia, affective anxiety, cognitive risk and reproductive appraisal, protective or avoidance-related behaviour, and organisational modifiers. A study could contribute to more than one domain without implying that the domains represented a single latent construct. Patterns were compared across populations, measurement approaches, and study designs, and contradictory findings and source-level inconsistencies were retained. Consistent with the mapping purpose of the review, no formal risk-of-bias score was used, and no study was excluded on the basis of methodological quality. Instead, study-level limitations are systematically charted in Table 2, including sampling and response rate, instrument validation, cross-sectional design, denominator ambiguity, non-random allocation, and short follow-up. These limitations affect confidence in the magnitude and generalisability of individual associations but do not negate their value for mapping constructs, measurement problems, and hypotheses.

## 3. Results

### 3.1. Search Results and Study Characteristics

**Figure 1:**
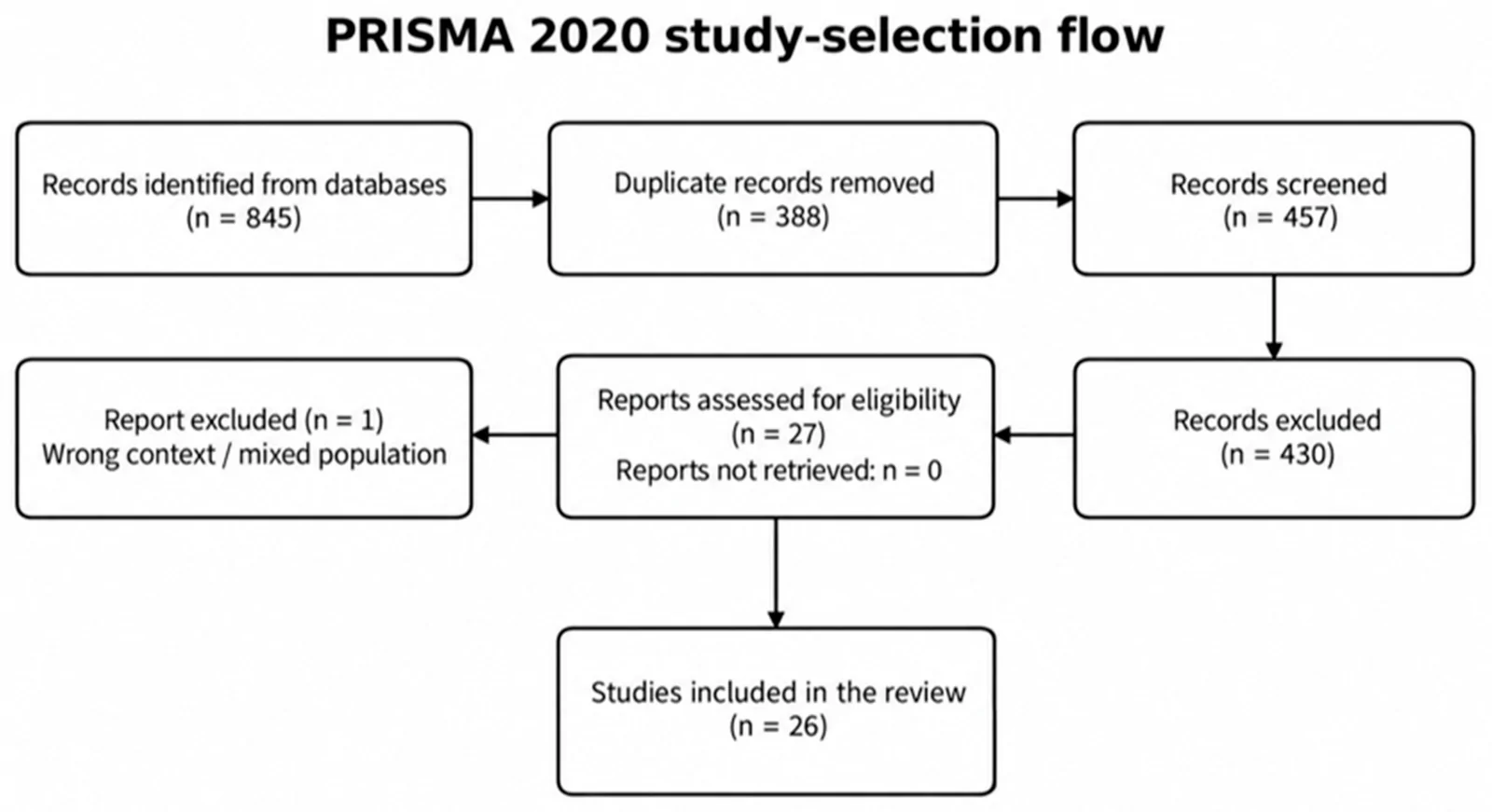
PRISMA 2020 study-selection flow for the updated search. Counts are consistent with Section 3.1 and Supplementary Tables S1 and S2.

The searches yielded 845 records. After removal of 388 duplicates, 457 records were screened by title and abstract; 430 were excluded and 27 reports were assessed in full text. One report was excluded because the population and radiation context were mixed and did not permit a healthcare-specific occupational interpretation (Supplementary Table S2). Twenty-six studies were included in the synthesis (Figure 1).

The 26 included studies were published from 2001 to 2026. Populations included nurses, radiographers, physicians in procedure-intensive specialties, military medical personnel, radiotherapy workers, and nursing, dental, radiography, and medical students. Cross-sectional surveys predominated. Educational evidence comprised pilot pre–post, quasi-experimental, repeated cross-sectional, augmented-reality, and longitudinal designs; one French cohort linked questionnaire responses to national dosimetry records, and mixed-methods research added contextual evidence (Table 2).

### 3.2. Direct Radiophobia and Affective Fear

Only a minority of studies explicitly used the term radiophobia. Pastel and Mulvaney measured radiation knowledge and attitudes before and after a three-day course; the mean attitude score shifted from 23 ± 9 to 18 ± 9, where lower scores represented more positive attitudes. The authors did not perform inferential comparisons and described the instrument as a pilot that had not been validated or tested for reliability [10]. Alotaibi and Saeed reported that 30 of 35 radiology nurses were afraid of radiation because of hazards such as cancer and infertility, while presenting 30 as 83%; this source-level arithmetic inconsistency is retained [11]. Ohno and Kaori reported that about 40% of 134 nurses had a fear of occupational-radiation exposure [12].

Ago et al. provided a direct source-labelled operationalisation: 174 of 274 valid survey respondents (63.5%) reported ever experiencing radiophobia during training, and 122 of these 174 (70.1%) reported first experiencing it in the first academic year [26]. The authors explicitly distinguished this occurrence/ever-experienced measure from prevalence and incidence. Shafiee et al. reported 17 nurses with radiophobia and withdrawal from fluoroscopy participation in four female nurses; however, the source reports 17/174 nurses (9.7%) in the original article’s Table 1 and later describes 17 nurses as 54.8%, creating an internal denominator inconsistency [17]. These findings support mapping of source-labelled fear constructs but do not provide a common prevalence estimate.

### 3.3. Occupational and Reproductive Concern

Study-level percentages in this section are reported descriptively to characterise individual samples and should not be interpreted as directly comparable prevalence estimates. Sampling methods, response rates, denominators, populations, and measurement instruments varied substantially across studies. Among paediatric anaesthesiologists, 76.8% of respondents were concerned or very concerned, women had higher concern than men (OR 1.66, 95% CI 1.20–2.31), and 73% (151/206) of respondents who had been pregnant during practice requested not to be assigned to X-ray cases; the overall survey response rate was 21% [15]. Concern was reported by 94% of neurosurgeon respondents, although the survey response rate was 6.8%; 59% did not use a dosimeter, and only 3% correctly identified the occupational exposure limit [21]. Among orthopaedic surgeon respondents, 75 of 85 (88.2%) reported worry about occupational effects, while 39 of 85 (45.9%) reported formal fluoroscopy training [24].

Pregnancy-focused studies demonstrated behavioural consequences. Female interventional radiologists more often changed practice because of exposure concern than men (23% vs. 13%), while 6% of pregnant interventional radiologists completely abstained from fluoroscopically guided interventions [13]. In the urology survey, women more often reduced hours or changed practice because of concern (17% vs. 7%, *p* = 0.01); 67% of previously pregnant respondents did not know their dosimetry dose [22]. Among European radiographers and students, 30% were concerned about health effects, 24% about fertility, and 19% about children’s health, with greater reproductive concern among younger respondents and students [25].

### 3.4. Education, Knowledge, and Risk Appraisal

Educational effects were not uniform. Japanese dental students showed decreased X-ray risk perception after education, whereas Indonesian students showed increased X-ray risk perception (both *p* < 0.05) [14]. Japanese nursing students ranked X-rays relatively highly among 30 hazards, and greater understanding was associated with lower risk perception, but cross-sectional analysis cannot determine whether knowledge changed perception [16]. In the longitudinal study by Cao et al., fear declined after coursework and declined further after onsite observation (both *p* < 0.001), alongside increased interest and career intention [20].

The quasi-experimental chest-equivalent-dose study included 71 nurses (Sievert-based education *n* = 40; CED-based education *n* = 31) and reported larger immediate improvements in radiation knowledge and larger reductions in radiation anxiety and radiation-related feelings in the CED group [27]. Because allocation was non-randomised and outcomes were measured immediately after education, the study supports a short-term association with communication format rather than long-term effectiveness. Wahab and Younis reported improved radiation-protection behaviour scores after a PMT-based educational intervention, but their article describes the study design inconsistently, warranting cautious interpretation [34].

### 3.5. Organisational Context and Behaviour

Baudin et al. showed that feeling personally exposed was associated with wearing a dosimeter (OR 3.69, 95% CI 2.04–6.66) and using protective practices (OR 4.60, 95% CI 2.28–9.30) [19]. The main reported barriers to protection were unsuitable or insufficient equipment. In Kazakhstan, occupational anxiety was associated with non-use of aprons, non-use of dosimeters, and inadequate safety training, although causal direction cannot be inferred [23].

The Ghanaian mixed-methods study linked students’ source-labelled radiophobia to misinformation, limited foundational knowledge, absence of personal monitoring badges, and inconsistent safety practice at placements [26]. European radiographers described shielding discomfort, limited availability, workflow pressure, and uncertainty about long-term low-dose effects [25]. These observations show that radiation concern may coexist with deficiencies in the working or training environment and should not be interpreted automatically as an irrational individual response (Table 3).

### 3.6. Additional Evidence on Communication, Training, and Occupational Consequences

Additional included studies added evidence on communication, training, and occupational consequences. Physicians ranked equivalent dose quantities differently depending on the language used to express them, while an augmented-reality-supported education study reported post-training changes in knowledge and risk rankings among nursing students [28,35]. High concern frequently coexisted with incomplete training or practice: 82% of anaesthesiology residents and 93% of interventional pain physicians reported substantial concern, while the resident dosimetry project measured low doses relative to occupational limits and the international pain-physician survey documented gaps in knowledge and practice [29,32]. These results describe co-occurring patterns rather than causal mechanisms.

Radiation anxiety among Chinese medical radiation workers was associated with burnout and intention to leave [33]. In Indian radiotherapy facilities, differences in perceived risk were reported alongside variation in implementation of regulatory requirements [30]. The operating-room pilot showed that ionising radiation was ranked as a major occupational risk, with weighting varying by specialty [31]. A PMT-based intervention reported post-training changes in protective behaviour among nurses [34]. Collectively, these findings justify distinguishing affective concern, cognitive appraisal, protective behaviour, and avoidance-related responses without assuming that one necessarily causes another.

### 3.7. Framework-Based Qualitative Synthesis

**Figure 2:**
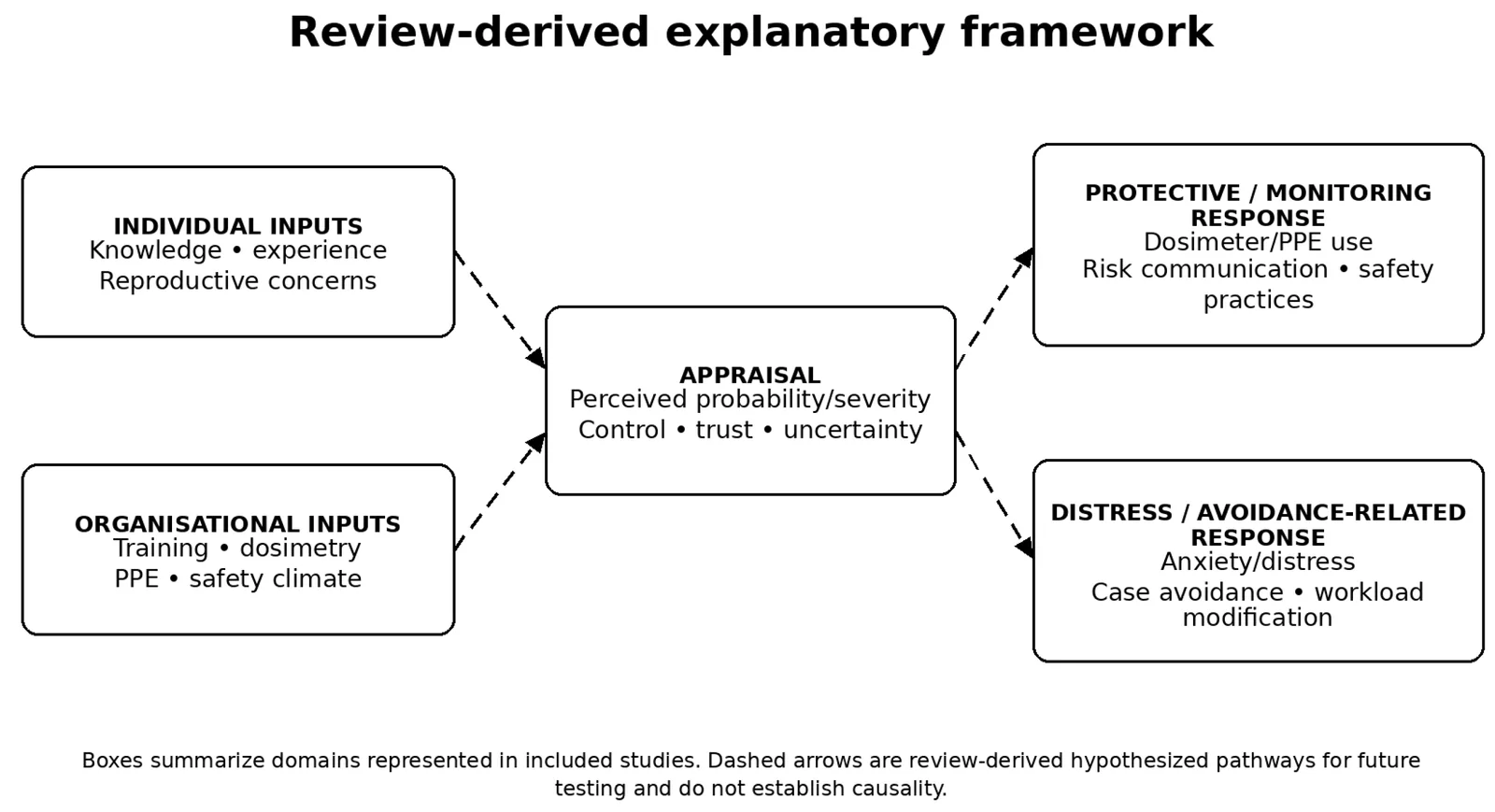
Review-derived conceptual framework. Boxes summarise domains represented in the included evidence; dashed arrows denote review-derived hypothesised pathways for future testing and do not establish causal relationships. Occupational responses are described as protective/monitoring or distress/avoidance-related rather than classified as adaptive or maladaptive.

A framework synthesis was applied to the charted findings. Evidence was coded into five linked but analytically distinct domains: direct fear/source-labelled radiophobia, affective anxiety, cognitive risk and reproductive appraisal, protective or avoidance-related behaviour, and organisational modifiers. The framework is a review-derived conceptual model intended to organise observed evidence and generate hypotheses for prospective testing; it is not a causal model. Organisational and individual domains are therefore shown as potential contextual inputs, and the pathways between appraisal and occupational responses are hypothesised rather than empirically established across the included studies (Table 4). The resulting review-derived conceptual framework is shown in Figure 2.

**Figure 3:**
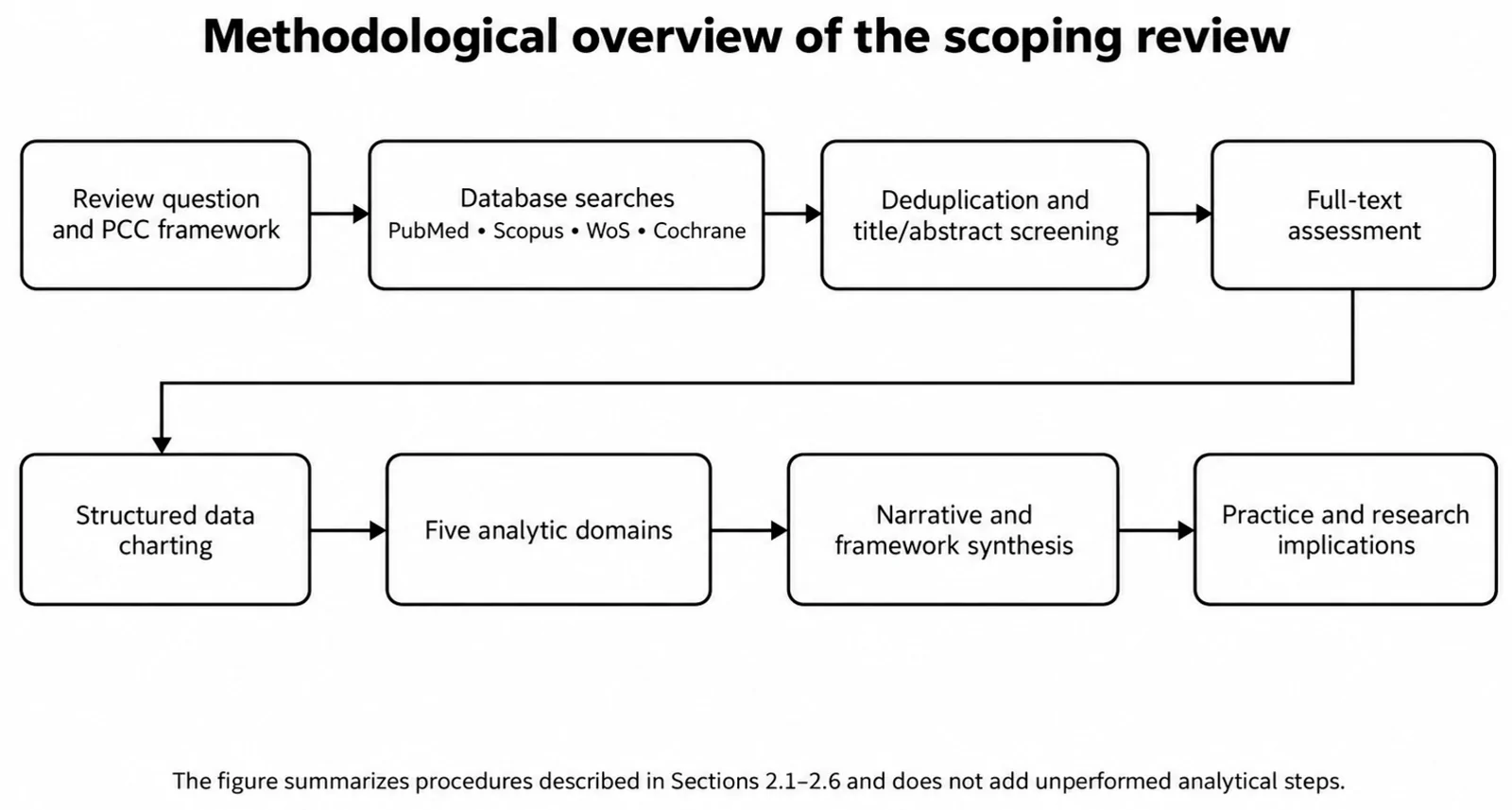
Methodological overview of the scoping review. The diagram summarises the procedures reported in Section 2.1, Section 2.2, Section 2.3, Section 2.4, Section 2.5 and Section 2.6 and does not introduce additional analytical steps.

For completeness, Figure 3 summarises the methodological workflow described in Section 2.1, Section 2.2, Section 2.3, Section 2.4, Section 2.5 and Section 2.6.

### 3.8. Patterns by Population and Clinical Context

Patterns differed qualitatively across populations and settings, although heterogeneity precluded formal subgroup meta-analysis. Student studies commonly examined knowledge, risk perception, first experiences of fear, or responses to education, whereas studies of practicing professionals more often addressed occupational concern, monitoring, protective practice, workload modification, or career consequences. Pregnancy-focused studies among interventional radiologists and urologists centred on reproductive concern, access to guidance and protective equipment, and changes in practice; these outcomes should not be conflated with generalised anxiety among students. Procedure-intensive specialties such as interventional radiology, anaesthesia, neurosurgery, orthopaedics, radiotherapy, and interventional pain medicine were represented, but exposure intensity could not be standardised because the studies did not use a common dosimetric metric. Sex/gender and pregnancy status were relevant in several reports, while younger participants or students showed greater reproductive concern in the European radiographer survey. Cross-sectional surveys predominated, so differences by profession, country, experience, or training level are descriptive patterns rather than evidence of causal subgroup effects.

## 4. Discussion

### 4.1. Principal Findings

This review identified 26 studies but no standard definition, validated diagnostic threshold, or common measurement instrument for professional radiophobia. The evidence encompasses analytically distinct constructs: source-labelled radiophobia or explicit fear, affective anxiety or worry, cognitive risk appraisal, reproductive concern, protective behaviour, and avoidance-related responses. The broad evidence-mapping scope does not assume that these constructs are manifestations of a single latent phenomenon; they are synthesised within a common occupational-radiation context while remaining conceptually distinct.

The wide numerical range in reported fear or concern should not be interpreted as variation in a single prevalence parameter. Study-level percentages describe individual samples and arose from different professions, exposure contexts, question wording, response options, time frames, denominators, and response rates. Ago et al. explicitly described their 63.5% result as occurrence/ever-experienced radiophobia rather than prevalence or incidence [26]. Alotaibi and Saeed [11] and Shafiee et al. [17] contain source-level numerical or denominator inconsistencies. Preserving these inconsistencies rather than silently recalculating them supports the conclusion that the evidence is heterogeneous and not suitable for pooled prevalence estimation.

### 4.2. Interpretation of Education and Experience

Education was generally associated with improved knowledge, but affective and risk-appraisal outcomes differed by content and context. A longitudinal student cohort reported reductions in radiation anxiety across educational time points [20], and a small quasi-experimental comparison reported larger immediate reductions in anxiety after symbolic CED communication than after Sievert-based teaching [27]. In contrast, X-ray risk perception increased after education in the Indonesian student group while decreasing in the Japanese group [14]. These observations do not establish a single educational effect. They suggest that future studies should distinguish knowledge acquisition from affective response and risk appraisal and should use prospective designs with longer follow-up.

Greater experience was associated in some studies with different knowledge or risk-appraisal patterns, but the reviewed evidence does not establish a causal protective effect. Most studies were cross-sectional, and experience is entangled with profession, training, age, selection into radiation-intensive work, and local safety arrangements. Longitudinal designs using validated instruments are needed before experience can be treated as an intervention mechanism.

### 4.3. Distinguishing Fear and Anxiety from Contextually Appropriate Risk Appraisal and Protective Behaviour

The included studies do not provide validated criteria for classifying radiation-related concern as disproportionate, justified, adaptive, or maladaptive. Accordingly, this review does not assign such labels to individual behaviours. Instead, it distinguishes measured affective fear or anxiety from cognitive risk appraisal, reproductive concern, protective practice, and avoidance-related responses, and interprets each in relation to the study context.

Lack of dosimetry feedback, unavailable or poorly fitting protection, inconsistent local practice, and unclear pregnancy policies provide documented contextual reasons for uncertainty [19,22,25,26]. Dosimeter use, shielding, or workflow modification may represent appropriate safety practice; conversely, avoidance or reduced participation may have different meanings depending on objective exposure, procedural requirements, available protection, and functional impact. The reviewed studies did not provide a common validated threshold with which to adjudicate these distinctions.

Figure 2 therefore presents organisational and individual conditions as review-derived contextual domains and uses hypothesised pathways only to organise the evidence. This is consistent with international occupational-radiation guidance emphasising monitoring, education, equipment, optimisation, and quality assurance, and with published work on radiation-safety climate [36,37,38], while the included observational evidence itself does not establish causal pathways.

### 4.4. Implications for Practice and Research

For practice, the evidence supports attention to dose and risk communication, practical radiation-protection training, individual monitoring and feedback, availability and usability of protective equipment, and clear mechanisms for discussing pregnancy and occupational exposure. Because the included studies were heterogeneous and predominantly observational, this review does not rank these components or establish their comparative effectiveness. Risk communication should accurately convey magnitude and uncertainty and support informed benefit-risk discussion [39].

The need for more coherent measurement follows directly from the heterogeneity observed across the included studies. A future multidimensional instrument could separate factual knowledge, perceived probability and severity, affective fear or anxiety, perceived controllability, trust in monitoring, protective behaviour, and avoidance. Requirements such as content validity, cognitive interviewing, structural validity, internal consistency, test–retest reliability, measurement invariance, and validated thresholds are methodological recommendations for future instrument development rather than empirical findings of the present review. Prospective studies should also consider objective dosimetry, training exposure, safety-climate measures, and observed behaviour.

The findings also have relevance for health systems in which diagnostic and interventional radiology services are expanding. Across the included studies, radiation-related concern was reported alongside potentially modifiable organisational factors, including gaps in training, dosimetry feedback, availability or usability of protective equipment, workflow constraints, and uncertainty regarding pregnancy-related practice [19,22,25,26,29,32]. Expansion of radiological services should therefore be accompanied by proportionate development of workforce radiation-safety training, individual monitoring and feedback, accessible protective equipment, and transparent risk communication. In Kazakhstan, available evidence similarly suggests that occupational anxiety may coexist with training gaps, inconsistent dosimetry use, and deficiencies in radiation-protection practices [23]. These implications are hypothesis-generating and should be evaluated prospectively in different health-system contexts.

### 4.5. Strengths and Limitations

Strengths include explicit PCC criteria, database-specific search reporting, preservation of source-level inconsistencies, conceptual separation of affective, cognitive, reproductive, behavioural, and organisational domains, and independent rechecking of key extracted data against the original reports during revision. Supplementary Table S4 records the verification status and source-specific qualifications for the 26 included studies.

Limitations are substantial. The English-language restriction may have introduced language bias. The search did not include CINAHL, PsycINFO, Embase, or a structured grey-literature search, and no formal backward/forward citation tracking or independent peer review of the search strategy was documented; relevant psychological, nursing, or other subject-specific evidence may therefore have been missed. This review is best understood as a structured map of evidence retrieved from the searched databases rather than an exhaustive census of all relevant literature. The protocol was not prospectively registered, the exact method-finalisation date was not recorded, and no archived public review plan is available. One reviewer performed the initial screening and charting; although potentially eligible and full-text decisions received secondary checking and all included-study extractions were independently rechecked during revision, the original extent of independent screening and disagreement frequency were not prospectively documented and no formal calibration exercise was recorded. Complete PubMed, Web of Science, and Cochrane exports and the record-level screening log were not retained, preventing full record-level reconstruction of deduplication and screening decisions. Most included studies were cross-sectional, convenience, single-centre, or low-response surveys; instruments and outcome wording differed, several measures were not validated, and intervention studies were small or had short follow-up. Formal risk-of-bias scoring was not undertaken. Consequently, the review maps constructs and generates hypotheses but does not estimate pooled prevalence, establish causality, or rank intervention effectiveness.

## 5. Conclusions

The literature does not support a standardised operational definition or common measurement framework for professional radiophobia. Included studies reported source-labelled radiophobia, radiation-related fear or anxiety, cognitive risk appraisal, reproductive concern, protective behaviour, and avoidance-related responses in different professional and student samples, but these constructs are not interchangeable and their frequencies cannot be pooled or generalised as a single prevalence estimate. Knowledge, education, experience, risk communication, dosimetry feedback, equipment usability, workflow, and safety climate were reported alongside these outcomes, but predominantly observational evidence does not establish causal pathways. Future research should distinguish radiation-related fear and anxiety from contextually appropriate occupational risk appraisal and protective behaviour, use validated multidimensional measures, and test review-derived hypotheses prospectively with objective exposure and organisational data.

## Figures and Tables

**Table 1:** Operational definitions used to organise the evidence map.

Construct	Operational Meaning in This Review	How It Is Represented in the Included Studies
Radiophobia	Source-used term for exaggerated or explicit fear of ionising radiation; not treated as a validated diagnosis.	Explicit self-identification, direct fear items, or source terminology using radiophobia.
Radiation-related fear	Affective fear attributed to ionising radiation or occupational exposure.	Fear or uneasiness items concerning radiation exposure.
Occupational anxiety	Worry or anxiety related to radiation work, exposure, or anticipated consequences.	Anxiety scales, worry/concern ratings, negative emotions.
Perceived radiation risk	Cognitive appraisal of probability, severity, or relative danger of radiation-related outcomes.	Risk ratings, ranking tasks, dose/risk communication responses.
Reproductive concern	Concern about fertility, pregnancy, foetal effects, or future-child health.	Pregnancy/fertility questions and practice changes attributed to reproductive concern.
Protective behaviour	Dose-reduction or monitoring actions that may be appropriate safety practice and are not evidence of phobia by themselves.	Dosimeter use, shielding, distance, workflow modification, safety practices.
Avoidance behaviour	Reduction, withdrawal, or refusal of radiation-related activity because of concern; not assumed to be pathological without contextual criteria.	Case avoidance, withdrawal from procedures, reduced workload or career-related avoidance.

**Table 2:** Characteristics and principal findings of the included studies.

Study	Population	Design/Construct	Key Reported Finding and Interpretive Limitation
Pastel and Mulvaney, 2001 [10]	USA; 72 pre-course and 59 post-course military medical personnel	Pilot pre–post RadKAS; knowledge and attitudes/fear	Attitude score shifted from 23 ± 9 to 18 ± 9 (lower = more positive); knowledge–attitude correlations were 0.677 before and 0.643 after training. No inferential comparison; instrument not validated.
Alotaibi and Saeed, 2006 [11]	Kuwait; 35 radiology nurses	Cross-sectional questionnaire; awareness and fear	The authors reported 30 nurses as afraid of radiation because of hazards such as cancer and infertility and presented this as 83%; with a sample of 35, the percentage is internally inconsistent. Small sample and source-level numerical inconsistency.
Ohno and Kaori, 2011 [12]	Japan; 134 nurses in five hospitals	Cross-sectional questionnaire; occupational fear and knowledge	About 40% reported fear of occupational exposure; nurses without radiological-practice experience showed poorer knowledge. The study used a questionnaire in 134 nurses with a 100% collection rate.
Ghatan et al., 2016 [13]	USA; 534 interventional radiologists	Cross-sectional survey; pregnancy concerns and practice modification	Women more often changed practice because of exposure concerns than men (23% vs. 13%); 6% of pregnant interventional radiologists abstained from fluoroscopically guided interventions. Response rate 10%.
Yoshida et al., 2019 [14]	Japan and Indonesia; 107 and 137 dental students	Repeated cross-sectional educational comparison; risk-perception ratings	After education, X-ray risk perception decreased in Japanese students but increased in Indonesian students (both *p* < 0.05), indicating context-dependent rather than uniformly reassuring effects.
Whitney et al., 2019 [15]	USA; 674 paediatric anaesthesiologists	Cross-sectional survey; concern, protective practices and avoidance	Overall, 76.8% were concerned/very concerned; women had higher concern (OR 1.66, 95% CI 1.20–2.31). Among respondents who had been pregnant during practice, 73% (151/206) requested avoidance of X-ray cases; response rate was 21%.
Yoshida et al., 2020 [16]	Japan; 242 nursing students (84.3% response)	Cross-sectional questionnaire; knowledge and seven-point risk perception	X-rays ranked fourth to ninth among 30 risks, higher than in two US comparison groups. Greater understanding was associated with lower risk perception, but the design was cross-sectional.
Shafiee et al., 2020 [17]	Iran; questionnaire *n* = 215 (174 nurses, 41 physicians); practice checklist *n* = 31	Questionnaire plus practice checklist; knowledge, practice and radiophobia item	Table 1 reported radiophobia in 17/174 nurses (9.7%) and the text reported withdrawal from fluoroscopy participation in four female nurses; the Discussion also described 17 nurses as 54.8%, creating a source-level denominator inconsistency.
Goula et al., 2021 [18]	Greece; 132 operating-room professionals	Single-centre cross-sectional survey; beliefs, emotions and behaviour	Younger and less experienced staff reported more negative emotions related to radiation; psychosomatic manifestations were also examined. Single-centre cross-sectional design and study-specific questionnaire limit generalisability.
Baudin et al., 2024 [19]	France; cohort 1457, questionnaire respondents 428	SISERI dosimetry linked to validated 29-item questionnaire	Feeling personally IR-exposed was associated with dosimeter wearing (OR 3.69, 95% CI 2.04–6.66) and radiation-protection practices (OR 4.60, 95% CI 2.28–9.30). Observational associations do not establish directionality.
Cao et al., 2024 [20]	China; 111 fourth-year undergraduates assessed at three time points	Longitudinal educational study; ordinal fear, interest and career intention	Fear of X-rays and interventional-radiology exposure fell after coursework and again after onsite observation (both *p* < 0.001); single cohort and self-report outcomes.
McCloskey et al., 2024 [21]	USA; 227 neurosurgeons (6.8% response)	National cross-sectional survey; concern, knowledge and practice	Concern was reported by 94%; 59% did not use a dosimeter and only 3% identified the occupational exposure limit. Low response raises selection-bias concerns.
Wenzel et al., 2024 [22]	International/primarily USA; 384 urologists	Cross-sectional mixed survey; concern, pregnancy practice and qualitative comments	Women more often decreased hours or changed practice because of radiation concern (17% vs. 7%, *p* = 0.01); among 164 previously pregnant respondents, 110 (67%) did not know their dosimeter dose. Survey respondents over-represented women and were predominantly U.S.-based.
Darbayeva et al., 2025 [23]	Kazakhstan; survey *n* = 324; dosimetry *n* = 177	Cross-sectional survey plus service and dosimetry data; Spielberger–Hanin scale	Thirty-two percent reported occupational-radiation anxiety; anxiety was associated with non-use of aprons, non-use of dosimeters and inadequate training. Cross-sectional associations do not establish direction.
Karavadra et al., 2025 [24]	United Kingdom; 85 orthopaedic surgeons	Single-trust cross-sectional survey; knowledge and worry	Seventy-five of 85 (88.2%) reported worry about occupational effects; 39/85 (45.9%) reported formal fluoroscopy training. Single-trust sample limits generalisability.
Brage et al., 2026 [25]	Europe; 629 radiographers and students from 32 countries	Cross-sectional mixed survey; occupational and reproductive concern	Concern was reported for health effects (30%), fertility (24%) and children’s health (19%); younger respondents and students expressed greater reproductive concern. Cross-sectional self-report data from 629 respondents in 32 countries.
Ago et al., 2026 [26]	Ghana; 18 interviews and 274 survey responses	Exploratory sequential mixed methods; direct radiophobia experience	Of 274 valid survey responses, 174 (63.5%) reported ever experiencing what the authors termed radiophobia; 122/174 (70.1%) reported first experiencing it in the first academic year. The authors explicitly described this as occurrence/ever-experienced rather than prevalence or incidence.
Ayasrah and Alrashdan, 2026 [27]	Jordan; 71 nurses (Sievert *n* = 40; CED *n* = 31)	Quasi-experimental pre–post comparison; RKQ, RAQ and RREF	The final sample comprised 71 nurses (Sievert group *n* = 40; CED group *n* = 31). CED-based education produced larger immediate improvements in RKQ and reductions in RAQ and RREF than Sievert-based education. Quasi-experimental, non-randomised comparison with immediate post-test outcomes.
Narayan et al., 2016 [28]	USA; 96 internal-medicine and paediatric residents	Cross-sectional online experiment; perceived dose/risk under alternative wording	Equivalent dose quantities were ranked differently depending on wording (*p* < 0.001); the ACR relative radiation level had the highest correct ranking (61.2%). Perception was inferred from ranking rather than an anxiety scale.
Wang et al., 2017 [29]	USA; concern survey *n* = 57; dosimetry project *n* = 41 anaesthesiology residents	Institutional survey plus short-term dosimetry; occupational concern	Eighty-two percent reported high/very high concern and 94% wanted education, while measured doses were low relative to limits. Single-centre survey and different samples for concern and dosimetry.
Singh, 2018 [30]	India; workers in nine radiotherapy facilities	Semi-structured and in-depth questionnaire; organisational risk perception	Risk perceptions varied across professional groups and were reported alongside variation in implementation of regulatory requirements. Facility-level qualitative reporting limits individual effect estimates.
Corvino et al., 2021 [31]	Italy; 50 postgraduate clinicians working in operating rooms	Cross-sectional pilot; Analytic Hierarchy Process occupational-risk weighting	Ionising radiation ranked second among perceived operating-room risks (w = 0.219); anaesthesiologists assigned the lowest ionising-radiation risk weight (w = 0.1657). Small single-centre pilot using an Analytic Hierarchy Process instrument.
Provenzano et al., 2021 [32]	International; 708 interventional pain physicians	Web survey; concern, training, knowledge, and practice	Ninety-three percent reported concern; 63% had received radiation-safety training. At least 80% compliance occurred for only 2/15 practices and for 0/10 knowledge questions. Voluntary professional-society survey.
Cui et al., 2022 [33]	China; 277 medical radiation workers	Cross-sectional survey; exposure anxiety, SOC-13, MBI-HSS, intention to leave	Radiation-exposure anxiety was associated with lower sense of coherence, higher burnout, and intention to leave employment; pregnancy-related caution was common. Cross-sectional design precludes directionality.
Wahab and Younis, 2026 [34]	Iraq; 60 radiation-exposed hospital nurses (30 intervention, 30 control)	Controlled pre–post PMT educational intervention; threat/coping appraisal and protective behaviour	Behaviour scores increased from 3.23 before training to 4.58 after the intervention and were 4.35 at the secondary assessment; the authors reported effect sizes of 0.88 for perceived severity and 0.78 for perceived susceptibility. The paper describes the design inconsistently as randomised quasi-experimental and as a true experimental randomised controlled trial, warranting cautious interpretation.
Matsumoto et al., 2026 [35]	Japan; 80 second-year nursing students	Pre–post AR plus lecture; knowledge and seven-point risk perception	Knowledge improved and rankings for X-rays and nuclear power decreased by one rank after training. Single-group immediate assessment cannot isolate the AR component or demonstrate durability.

Note: Alotaibi and Saeed (2006) [11] reported 30 of 35 nurses as 83%; however, 30/35 corresponds to 85.7%. Shafiee et al. (2020) [17] reported 17 nurses with radiophobia as 9.7% in the original article’s Table 1 but later described 17 nurses as 54.8% in the Discussion section of the source article, indicating a second source-level denominator inconsistency. These discrepancies are retained and identified rather than retrospectively corrected.

**Table 3:** Conceptual domains represented across the included studies.

Domain	Contributing Studies	How the Domain Is Represented
Direct fear/radiophobia	Pastel; Alotaibi and Saeed; Ohno and Kaori; Shafiee; Ago	Explicit fear items, attitude scales, self-identification, or reported radiophobia
Affective anxiety/worry	Goula; Cao; McCloskey; Darbayeva; Karavadra; Ayasrah and Alrashdan; Provenzano; Cui; Wang	Anxiety, negative emotion, worry, or concern about occupational exposure
Risk/reproductive appraisal	Ghatan; Yoshida (dental); Whitney; Yoshida (nursing); Wenzel; Brage; Narayan; Corvino; Wahab and Younis; Matsumoto	Perceived risk, cancer/fertility concern, pregnancy-related concern
Protective/avoidant behaviour	Ghatan; Whitney; Shafiee; Baudin; Wenzel; Darbayeva; Provenzano; Cui; Wahab and Younis	Dosimeter or PPE use, changes in workload, case avoidance or procedure withdrawal
Organisational modifiers	Goula; Baudin; Wenzel; Darbayeva; Brage; Ago; Singh; Provenzano; Wang	Training access, dosimetry feedback, equipment availability/fit, workflow and safety climate

**Table 4:** Framework synthesis of professional radiation concern and occupational responses.

Analytic Theme	Evidence Pattern	Interpretation	Practice Implication
Construct fragmentation	Fear, anxiety, worry, risk perception, and avoidance were measured with different items and scales.	Reported proportions are not directly comparable; a pooled prevalence would be misleading.	Use a multidimensional, validated instrument and report subscales separately.
Knowledge–emotion gap	Education often improved knowledge, but effects on anxiety and behaviour were inconsistent.	Changes in knowledge were not consistently accompanied by parallel changes in anxiety or behaviour.	Future interventions should evaluate dose literacy alongside practical rehearsal, feedback, and dialogue.
Protective and avoidance-related responses	Concern was reported alongside both dosimeter/PPE use and reduced workload or procedure avoidance.	The review did not apply a post hoc validated threshold for classifying behaviour as adaptive or maladaptive; interpretation depends on measured exposure, available protection, procedural requirements, and functional impact.	Describe observed behaviour and its context; evaluate proportionality and functional impact in future prospective studies.
Reproductive and career concerns	Pregnancy, fertility, and future-child health concerns recurred across specialties and students.	Perceived stakes are amplified when dose information and pregnancy policies are unclear.	Provide preconception/pregnancy counselling and transparent dose monitoring.
Organisational context	Equipment fit/availability, dosimetry, training access, and safety climate were reported alongside different occupational response patterns.	Concern may reflect perceived or actual system limitations as well as individual appraisal.	Assess system-level safety conditions before interpreting concern as an individual deficit.

## Data Availability

The data supporting this scoping review are presented in the article and its Supplementary Materials. Supplementary Table S4 provides the final included-study verification dataset. The retained final Scopus RIS and CSV exports are available from the corresponding author on reasonable request. Complete PubMed, Web of Science, and Cochrane exports and the original record-level screening log were not retained; therefore, aggregate selection counts can be reconciled arithmetically, but record-level deduplication and screening decisions cannot be independently reconstructed in full.

## Supplementary Materials

The following supporting information can be downloaded at: https://www.mdpi.com/article/10.3390/healthcare14193353/s1, Table S1: Database-Specific Search Strategies; Table S2: Full-Text Report Excluded from the Review; Table S3: Completed checklist for the submitted manuscript; Table S4: Included-Study Data Verification Dataset and Source-Specific Qualifications.
