# Supplementary material for: Radiation-Related Fear, Risk Perception, and Occupational Responses Among Healthcare Workers and Health Professions Students: A Scoping Review
Source: Healthcare (Basel). 2026 Oct 8;14(19):3353. doi: 10.3390/healthcare14193353 (PMC13664887; doi:10.3390/healthcare14193353)
Supplement: Supplementary file 1 [file healthcare-14-03353-s001.zip › Table_S1.pdf]

Supplementary Materials

Radiation-Related Fear, Risk Perception, and Occupational Responses Among Healthcare Workers and Health Professions Students: A Scoping Review

Table S1. Database-Specific Search Strategies

The database-reported searches yielded 845 records before deduplication. After the results were combined, 388 duplicate records were removed (355 by identical DOI and 33 by identical normalised title), leaving 457 unique records for title and abstract screening. The final Scopus RIS and CSV exports each contained 359 records; a partial 90-record Scopus export from 3 September 2026 was an intermediate subset and was not added to the total. The aggregate review flow is arithmetically consistent: 235 + 359 + 243 + 8 = 845; 845 - 388 = 457; 457 - 430 = 27; and 27 - 1 = 26. Complete exports from PubMed, Web of Science, and Cochrane and the record-level screening log were not retained; consequently, this reconciliation confirms aggregate consistency but does not constitute independent record-level reconstruction.

Database, platform, and yield	Date searched	Coverage and limits	Search strategy and filters applied
PubMed (n = 235)	3 September 2026	1 January 2000-3 September 2026; English	(radiophobia[tiab] OR "fear of radiation"[tiab] OR "radiation fear"[tiab] OR "radiation anxiety"[tiab] OR "radiation-related anxiety"[tiab] OR "radiation risk perception"[tiab] OR "perceived radiation risk"[tiab] OR ((fear*[tiab] OR anxi*[tiab] OR concern*[tiab] OR "risk perception"[tiab]) AND ("ionizing radiation"[tiab] OR "ionising radiation"[tiab]))) AND ("Health Personnel"[Mesh] OR "Students, Health Occupations"[Mesh] OR "healthcare worker"[tiab] OR "healthcare workers"[tiab] OR "health care worker"[tiab] OR "health care workers"[tiab] OR "medical personnel"[tiab] OR physician*[tiab] OR doctor*[tiab] OR nurse*[tiab] OR radiographer*[tiab] OR technologist*[tiab] OR "medical student"[tiab] OR "medical students"[tiab] OR "nursing student"[tiab] OR "nursing students"[tiab] OR "radiography student"[tiab] OR "radiography students"[tiab]) AND ("2000/01/01"[Date - Publication] : "2026/09/03"[Date - Publication]) AND English[Language]
Scopus (n = 359)	4 September 2026	2000-2026; English	TITLE-ABS-KEY(radiophobia OR "fear of radiation" OR "radiation fear" OR "radiation anxiety" OR "radiation-related anxiety" OR "radiation risk perception" OR "perceived radiation risk" OR ((fear* OR anxi* OR concern* OR "risk perception") AND ("ionizing radiation" OR "ionising radiation"))) AND TITLE-ABS-KEY("healthcare worker" OR "healthcare workers" OR "health care worker" OR "health care workers" OR "medical personnel" OR physician* OR doctor* OR nurse* OR radiographer* OR technologist* OR "medical student" OR "medical students" OR "nursing student" OR "nursing students" OR "radiography student" OR "radiography students") AND

**Database, platform, and yield**	**Date searched**	**Coverage and limits**	**Search strategy and filters applied**
			PUBYEAR > 1999 AND PUBYEAR < 2027 AND LIMIT-TO(LANGUAGE, "English")
Web of Science Core Collection; Editions: All (n = 243)	4 September 2026	Publication years 2000-2026; English	TS=(radiophobia OR "fear of radiation" OR "radiation fear" OR "radiation anxiety" OR "radiation-related anxiety" OR "radiation risk perception" OR "perceived radiation risk" OR ((fear* OR anxi* OR concern* OR "risk perception") AND ("ionizing radiation" OR "ionising radiation"))) AND TS=("healthcare worker" OR "healthcare workers" OR "health care worker" OR "health care workers" OR "medical personnel" OR physician* OR doctor* OR nurse* OR radiographer* OR technologist* OR "medical student" OR "medical students" OR "nursing student" OR "nursing students" OR "radiography student" OR "radiography students") Filters applied in the results interface: Publication Years 2000-2026; Languages: English.
Cochrane Library (CENTRAL) (n = 8)	4 September 2026	January 2000-September 2026; English-language search terms	#1 (radiophobia OR "fear of radiation" OR "radiation fear" OR "radiation anxiety" OR "radiation-related anxiety" OR "radiation risk perception" OR "perceived radiation risk" OR ((fear* OR anxi* OR concern* OR "risk perception") AND ("ionizing radiation" OR "ionising radiation"))):ti,ab,kw [57] #2 ("healthcare worker" OR "healthcare workers" OR "health care worker" OR "health care workers" OR "medical personnel" OR physician* OR doctor* OR nurse* OR radiographer* OR technologist* OR "medical student" OR "medical students" OR "nursing student" OR "nursing students" OR "radiography student" OR "radiography students"):ti,ab,kw [126,673] #3 #1 AND #2; Cochrane Library publication date Jan 2000-Sep 2026 [8]
Total (n = 845)	-	-	Total records identified before deduplication
