# Supplementary material for: Radiation-Related Fear, Risk Perception, and Occupational Responses Among Healthcare Workers and Health Professions Students: A Scoping Review
Source: Healthcare (Basel). 2026 Oct 8;14(19):3353. doi: 10.3390/healthcare14193353 (PMC13664887; doi:10.3390/healthcare14193353)
Supplement: Supplementary file 1 [file healthcare-14-03353-s001.zip › Table_S2.pdf]

Supplementary Materials

Radiation-Related Fear, Risk Perception, and Occupational Responses Among Healthcare Workers and Health Professions Students: A Scoping Review

Table S2. Full-Text Report Excluded from the Review

Twenty-seven reports were assessed in full text. One report was excluded, and 26 studies were included in the scoping review.

Report	Population/context	Decision	Primary reason
Mihai, L.T.; Milu, C.; Voicu, B.; Enachescu, D. Ionizing radiation—understanding and acceptance. Health Phys. 2005, 89, 375–382. https://doi.org/10.1097/01.HP.0000175159.90986.E9	Mixed radiation workers, non-exposed physicians, and general population; included nuclear-energy and diagnostic-radiation perceptions	Excluded	Wrong context/mixed population: outcomes could not be restricted to healthcare workers' occupational or clinical medical-radiation fear.
