# Supplementary material for: Radiation-Related Fear, Risk Perception, and Occupational Responses Among Healthcare Workers and Health Professions Students: A Scoping Review
Source: Healthcare (Basel). 2026 Oct 8;14(19):3353. doi: 10.3390/healthcare14193353 (PMC13664887; doi:10.3390/healthcare14193353)
Supplement: Supplementary file 1 [file healthcare-14-03353-s001.zip › Table_S3.pdf]

PRISMA ScR Reporting Checklist

Radiation-Related Fear, Risk Perception, and Occupational Responses Among Healthcare Workers and Health Professions Students: A Scoping Review

Table S3. Completed checklist for the submitted manuscript

Section	Topic	Item	PRISMA-ScR checklist item	Reported on page number
TITLE	Title	1	Identify the report as a scoping review.	Page 1
ABSTRACT	Structured summary	2	Provide a structured summary that includes (as applicable): background, objectives, eligibility criteria, sources of evidence, charting methods, results, and conclusions that relate to the review questions and objectives.	Page 1
INTRODUCTION	Rationale	3	Describe the rationale for the review in the context of what is already known. Explain why the review questions/objectives lend themselves to a scoping review approach.	Pages 2-3
INTRODUCTION	Objectives	4	Provide an explicit statement of the questions and objectives being addressed with reference to their key elements (e.g., population or participants, concepts, and context) or other relevant key elements used to conceptualize the review questions and/or objectives.	Pages 2-4
METHODS	Protocol and registration	5	Indicate whether a review protocol exists; state if and where it can be accessed (e.g., a Web address); and if available, provide registration information, including the registration number.	Page 4; protocol not prospectively registered
METHODS	Eligibility criteria	6	Specify characteristics of the sources of evidence used as eligibility criteria (e.g., years considered, language, and publication status), and provide a rationale.	Page 5
METHODS	Information sources*	7	Describe all information sources in the search (e.g., databases with dates of coverage and contact with authors to identify additional sources), as well as the date the most recent search was executed.	Page 5 and Table S1

Section	Topic	Item	PRISMA-ScR checklist item	Reported on page number
METHODS	Search	8	Present the full electronic search strategy for at least 1 database, including any limits used, such that it could be repeated.	Table S1
METHODS	Selection of sources of evidence†	9	State the process for selecting sources of evidence (i.e., screening and eligibility) included in the scoping review.	Page 6; reviewer roles and secondary checking reported
METHODS	Data charting process‡	10	Describe the methods of charting data from the included sources of evidence (e.g., calibrated forms or forms that have been tested by the team before their use, and whether data charting was done independently or in duplicate) and any processes for obtaining and confirming data from investigators.	Page 6; charting fields and reviewer role reported; no formal pilot-testing/calibration documented; investigators not contacted; post-review independent recheck reported
METHODS	Data items	11	List and define all variables for which data were sought and any assumptions and simplifications made.	Page 6
METHODS	Critical appraisal of individual sources of evidence§	12	If done, provide a rationale for conducting a critical appraisal of included sources of evidence; describe the methods used and how this information was used in any data synthesis (if appropriate).	Not applicable; no formal risk-of-bias score was performed. Rationale and structured limitation charting on pages 6-7
METHODS	Synthesis of results	13	Describe the methods of handling and summarizing the data that were charted.	Pages 6-7

Section	Topic	Item	PRISMA-ScR checklist item	Reported on page number
RESULTS	Selection of sources of evidence	14	Give numbers of sources of evidence screened, assessed for eligibility, and included in the review, with reasons for exclusions at each stage, ideally using a flow diagram.	Pages 7-8, Figure 1, and Table S2
RESULTS	Characteristics of sources of evidence	15	For each source of evidence, present characteristics for which data were charted and provide the citations.	Pages 8-12, Table 2
RESULTS	Critical appraisal within sources of evidence	16	If done, present data on critical appraisal of included sources of evidence (see item 12).	Not applicable; no formal critical appraisal score was performed
RESULTS	Results of individual sources of evidence	17	For each included source of evidence, present the relevant data that were charted that relate to the review questions and objectives.	Pages 8-19, Table 2 and narrative Results
RESULTS	Synthesis of results	18	Summarize and/or present the charting results as they relate to the review questions and objectives.	Pages 13-19, Tables 3-4, and Figures 2-3
DISCUSSION	Summary of evidence	19	Summarize the main results (including an overview of concepts, themes, and types of evidence available), link to the review questions and objectives, and consider the relevance to key groups.	Pages 20-22
DISCUSSION	Limitations	20	Discuss the limitations of the scoping review process.	Page 22
DISCUSSION	Conclusions	21	Provide a general interpretation of the results with respect to the review questions and objectives, as well as potential implications and/or next steps.	Page 23
FUNDING	Funding	22	Describe sources of funding for the included sources of evidence, as well as sources of funding for the scoping review. Describe the role of the funders of the scoping review.	Page 24; the review received no external funding; funding of

Section	Topic	Item	PRISMA-ScR checklist item	Reported on page number
				included sources was not systematically charted

Source: Tricco AC, Lillie E, Zarin W, O'Brien KK, Colquhoun H, Levac D, et al. PRISMA Extension for Scoping Reviews (PRISMA-ScR): Checklist and Explanation. Ann Intern Med. 2018;169:467-473. doi:10.7326/M18-0850. Checklist wording reproduced from the official PRISMA-ScR checklist.

* Information sources are the places from which evidence is compiled, such as bibliographic databases and websites. † Sources of evidence is an inclusive term for eligible evidence or data sources. ‡ Data extraction in a scoping review is referred to as data charting. § Critical appraisal is the systematic examination of evidence validity, results, and relevance. The aggregate flow is arithmetically consistent; record-level reconstruction is not claimed because complete exports and the screening log were not retained.
