# Supplementary material for: Radiation-Related Fear, Risk Perception, and Occupational Responses Among Healthcare Workers and Health Professions Students: A Scoping Review
Source: Healthcare (Basel). 2026 Oct 8;14(19):3353. doi: 10.3390/healthcare14193353 (PMC13664887; doi:10.3390/healthcare14193353)
Supplement: Supplementary file 1 [file healthcare-14-03353-s001.zip › Table_S4.pdf]

Supplementary Table S4. Included-Study Data Verification Dataset and Source-Specific Qualifications

Purpose: to document the post-peer-review verification of key numerical and descriptive extractions for all 26 included studies. A second author independently rechecked the key extractions against the original reports. Source-level inconsistencies were retained and identified rather than silently recalculated. Verification does not constitute a formal risk-of-bias assessment.

Study	Verification status	Source-specific qualification / action in review
Pastel and Mulvaney, 2001 [10]	Verified against original report	Pre/post sample sizes, attitude scores, and knowledge-attitude correlations rechecked. The authors described the study as a pilot; the survey had not been validated or tested for reliability, and no inferential pre/post comparison was performed.
Alotaibi and Saeed, 2006 [11]	Verified; source-level inconsistency	Original report states that 30 of 35 nurses were afraid of radiation and presents this as 83%. The arithmetic is internally inconsistent (30/35 = 85.7%); the published value is retained and the discrepancy is explicitly noted.
Ohno and Kaori, 2011 [12]	Verified against original report	Sample n=134 with 100% questionnaire collection. The source states that about 40% of nurses had fear of occupational radiation exposure; this is retained as an approximate source-reported figure.
Ghatan et al., 2016 [13]	Verified against original report	n=534 respondents; 10% response rate. Women more often changed work practice because of occupational-exposure concerns than men (23% vs 13%); four pregnant interventional radiologists (6%) completely abstained from fluoroscopically guided interventions.
Yoshida et al., 2019 [14]	Verified against original report	Japanese and Indonesian dental-student samples and the opposite-direction changes in X-ray risk perception after lectures were rechecked. Findings are reported as context-dependent changes rather than a universal educational effect.
Whitney et al., 2019 [15]	Verified against original report	674/3151 (21%) response; 76.8% concerned/very concerned; women had higher concern (OR 1.66, 95% CI 1.20-2.31); 151/206 (73%) respondents who had been pregnant requested avoidance of X-ray cases.
Yoshida et al., 2020 [16]	Verified against original report	242/287 respondents (84.3%). Risk perception was measured for 30 events. The relationship between understanding and risk perception is reported as an association; the cross-sectional design does not establish direction.
Shafiee et al., 2020 [17]	Verified; source-level denominator inconsistency	The report identifies 17 nurses with radiophobia and four female nurses withdrawing from fluoroscopy participation. The source presents inconsistent denominators/percentages for the radiophobia result across sections; the review therefore reports the count and flags the inconsistency rather than deriving a new prevalence estimate.
Goula et al., 2021 [18]	Verified against original report	n=132. Results are described using the source terminology of negative emotions/psychosomatic manifestations and are not recast as a psychiatric diagnosis.
Baudin et al., 2024 [19]	Verified against original report	Feeling personally IR-exposed was associated with dosimeter wearing (OR 3.69, 95% CI 2.04-6.66) and radiation-protection practices (OR 4.60, 95% CI 2.28-9.30). Causal/predictive wording was avoided.
Cao et al., 2024 [20]	Verified against original report	The study included 111 students assessed at three time points. Repeated questionnaire observations are not presented as 333 independent participants.
McCloskey et al., 2024 [21]	Verified against original report	227/3344 (6.8%) responded; 134/227 (59%) did not use a dosimeter; 7/227 (3%) identified the occupational limit; 213/227 (94%) reported concern. Low response rate is retained as an interpretive limitation.
Wenzel et al., 2024 [22]	Verified against original report	n=384 overall; 164 respondents had previously been pregnant. Reduced hours/changed practice because of radiation concern was reported by 17% of women versus 7% of men (p=0.01); 110/164 (67%) did not know their dosimeter dose.
Darbayeva et al., 2025 [23]	Verified against original report	Survey n=324 and dosimetry n=177. Occupational radiation anxiety and radiation-protection practices are reported as cross-sectional associations; no causal direction is inferred.
Karavadra et al., 2025 [24]	Verified against original report	n=85; 39/85 (45.9%) reported formal fluoroscopy training and 75/85 (88.2%) reported worry about occupational health effects. Single-centre sampling remains a limitation.
Brage et al., 2026 [25]	Verified against original report	n=629 radiographers/students from 32 countries. Health, fertility, and future-child concerns and age/student patterns were rechecked against the original report. Results are reported as study-specific concern frequencies, not pooled prevalence.
Ago et al., 2026 [26]	Verified against original report	Of 274 valid responses, 174 (63.5%) reported ever experiencing radiophobia; 122/174 (70.1%) reported first experiencing it in the first academic year. The authors explicitly describe occurrence/ever-experienced radiophobia rather than prevalence or incidence.
Ayasrah and Alrashdan, 2026 [27]	Verified against original report	Total n=71; the CED and Sievert education groups and RKQ/RAQ/RREF outcomes were rechecked. Bibliographic citation uses volume 18 and article identifier 601232. Immediate between-group changes are not generalized beyond the study.
Narayan et al., 2016 [28]	Verified against original report	n=96 residents. Equivalent dose quantities were ranked differently depending on wording (p<0.001); the ACR relative radiation level had the highest correct ranking (61.2%). This is treated as risk/dose appraisal rather than anxiety.
Wang et al., 2017 [29]	Verified against original report	Concern survey n=57; 82% reported high/very high concern and 94% wanted education. The separate dosimetry component involved 41 residents; survey and dosimetry samples are kept distinct.

Study	Verification status	Source-specific qualification / action in review
Singh, 2018 [30]	Verified against original report	Workers from nine radiotherapy facilities were included. Differences in risk perception are reported alongside variation in implementation of regulatory requirements; the review avoids stronger causal wording.
Corvino et al., 2021 [31]	Verified against original report	n=50 postgraduate clinicians. Ionising radiation was weighted as a major operating-room risk (0.219), with the lowest specialty-specific radiation-risk weight reported for anesthesiologists (0.1657). The study is a small single-centre pilot using a broad occupational-risk instrument.
Provenzano et al., 2021 [32]	Verified against original report	n=708. 93% reported concern and 63% prior radiation-safety training. At least 80% compliance occurred for only 2/15 practice items and for 0/10 knowledge items.
Cui et al., 2022 [33]	Verified against original report	n=277. Radiation-exposure anxiety was associated with lower sense of coherence, higher burnout, and intention to leave; the cross-sectional design precludes causal direction.
Wahab and Younis, 2026 [34]	Verified against original report	30 intervention and 30 control participants. Protective-behaviour score was 3.23 pre-intervention, 4.58 post-intervention, and 4.35 at secondary testing. The authors label 0.88 (perceived severity) and 0.78 (perceived susceptibility) as effect sizes. The paper uses inconsistent design terminology (randomized quasi-experimental vs true experimental randomized controlled trial), so interpretation remains cautious.
Matsumoto et al., 2026 [35]	Verified against original report	n=80 second-year nursing students. Knowledge improved after AR-supported education and X-ray/nuclear-power risk rankings decreased by one rank. The single-group immediate pre/post design cannot isolate the AR component or establish durability.
